# Comparison of metabolic syndrome prevalence and characteristics using five different definitions in China: a population-based retrospective study

**DOI:** 10.3389/fpubh.2024.1333910

**Published:** 2024-02-19

**Authors:** Keli Ma, Haiyang Liu, Leilei Guo, Jinlong Li, Yunxiao Lei, Xiaoping Li, Lu Sun, Liu Yang, Ting Yuan, Congzhi Wang, Dongmei Zhang, Jing Li, Mingming Liu, Ying Hua, Lin Zhang

**Affiliations:** ^1^Department of Graduate School, Wannan Medical College, Wuhu, Anhui, China; ^2^Student Health Center, Wannan Medical College, Wuhu, Anhui, China; ^3^Department of Surgical Nursing, School of Nursing, Jinzhou Medical University, Jinzhou, Liaoning, China; ^4^Key Laboratory of Occupational Health and Safety for Coal Industry in Hebei Province, Department of Occupational and Environmental Health, School of Public Health, North China University of Science and Technology, Tangshan, Hebei, China; ^5^Obstetrics and Gynecology Nursing, School of Nursing, Wannan Medical College, Wuhu, Anhui, China; ^6^Department of Emergency and Critical Care Nursing, School of Nursing, Wannan Medical College, Wuhu, Anhui, China; ^7^Department of Internal Medicine Nursing, School of Nursing, Wannan Medical College, Wuhu, Anhui, China; ^8^Department of Pediatric Nursing, School of Nursing, Wannan Medical College, Wuhu, Anhui, China; ^9^Department of Surgical Nursing, School of Nursing, Wannan Medical College, Wuhu, Anhui, China; ^10^Rehabilitation Nursing, School of Nursing, Wannan Medical College, Wuhu, Anhui, China

**Keywords:** metabolic syndrome, prevalence, characteristics, China, retrospective study

## Abstract

**Background:**

Metabolic syndrome (MetS) is on the rise in developing countries and is characterized by a series of indications of metabolic disturbance. However, the prevalence of MetS varies under different definitions. The study aimed to compare five definitions of MetS in the China adult population, to explore their prevalence, characteristics and agreement.

**Methods:**

The data for the retrospective study came from the China Health and Retirement Longitudinal Study (CHARLS), consisting of 9,588 participants (≥45). MetS definitions from International Diabetes Federation (IDF) (2006), National Cholesterol Education Program Adult Treatment Panel III (ATPIII) (2005), National Cholesterol Education Program Adult Treatment Panel III (ATPIII) (2001), Chinese Diabetes society (CDS) (2004) and the World Health Organization (WHO) (1999). We used binary and multivariable logistic analysis to explore factors connected with MetS.

**Results:**

The five definitions of MetS led to different prevalence of MetS:34.52% by IDF (2006), 38.63% by ATP (2005), 25.94% by ATP (2001), 26.31% by CDS (2004), 21.57% by WHO (1999). According to the definition of IDF (2006) (22.32% vs. 45.06%), ATPIII (2005) definition (27.99% vs. 47.82%), ATPIII (2001) definition (15.37% vs. 35.07%), CDS (2004) definition (19.96% vs. 31.80%), and WHO (1999) definition (17.44% vs. 25.14%), the prevalence of MetS in men was low but in women was high. The agreement between the five definitions for men was good except for the IDF (2006) definition and ATPIII (2001) definition (kappa = 0.51), with kappa values from 0.64 to 0.85. For women, the agreement between the five definitions was good ranging from 0.67 to 0.95, however, except for the definition of CDS (2004) and the definition of IDF (2006) (kappa = 0.44), the definition of WHO (1999) and the definition of IDF (2006) (kappa = 0.55), and the definition of WHO (1999) and the definition of ATPIII (2005) (kappa = 0.54). Binary logistic analysis indicated that although the impact and relevance varied by sex and definition, age, education, marital status, current residence, current smoking, alcohol using, taking activities and number of chronic diseases were factors connected to MetS.

**Conclusion:**

the prevalence and characteristics of the five definitions of MetS are different in the Chinese population. Therefore, it is vital to use the same definition for a country to diagnose MetS. On the other side, a lower prevalence in men than in women and the consistency of five MetS definitions are good in men but relatively poor in women.

## Background

Metabolic syndrome (MetS) is a cluster of syndromes consisting of a variety of abnormal metabolic conditions associated with cardiovascular diseases (CVDs), including insulin resistance, elevated blood pressure and dyslipidemia (1, 2). Metabolic syndrome, the incidence of which continues to rise in developing countries, is a serious public health problem of increasing global importance (3). Approximately one quarter of the world’s population suffers from MetS (4). MetS consists mainly of central or abdominal obesity, diabetes mellitus, hypertension, and dyslipidemia characterized by hypertriglyceridemia (TG) and low high-density lipoprotein cholesterol (HDL-C) disorders. The mortality rate for cardiovascular events is higher in patients with MetS (5–7). Therefore, exploring the prevalence, characteristics and associated factors of metabolic syndrome is important for the prevention of cardiovascular diseases (CVDs) and slowing the increase in prevalence (8–10).

Currently there are different diagnostic criteria for MetS globally, in which the diagnostic criteria vary. In this study, the definitions of MetS provided by several international organizations were examined. The International Diabetes Federation (IDF) proposed a new definition in 2006 (11). The World Health Organization (WHO) proposed a definition of metabolic syndrome in 1999 (12). The definition published by the Adult Treatment Panel III (ATPIII) of the US National Cholesterol Education Program in 2001 was updated in 2005 (13, 14). The Chinese Diabetes Society (CDS) also proposed a definition of MetS in 2004 (15).

The prevalence of MetS varies globally according to different definitions, populations and geographical locations (16). According to studies, the global prevalence of MetS ranges from 12.5 to 31.4% (17). In China, the prevalence varies from 9.82 to 48.8% according to different criteria (16), and according to criteria of ATP III, the prevalence of MetS is 24.6% in men and 23.8% in women (18). In Peru, the prevalence of MetS was 30% according to a cross-sectional study by the Institute of Diabetes (IDF) (19), and in the United States, 32.8% of men and 36.6% of women had MetS according to ATP III 2011–2012 (20). This shows that the prevalence of MetS varies in the same country even when the same criteria are used. Thus, it is important to study the prevalence of MetS under different definitions, which may help researchers better define MetS.

## Methods

### Design and study participants

The data for this study came from the 2011 China Health and Retirement Longitudinal Survey wave (CHARLS Wave2011) conducted by the China Center for Economic Research at Peking University, a nationally representative longitudinal investigation (21, 22). This study area was conducted in 28 provinces and cities across China. The survey covered 450 villages or communities in 150 counties and districts. Subsequently, we collected data of metabolic syndrome for 2015, and the baseline data was chosen to be 2011 in CHARLS Wave 2011, We excluded individuals who met any of the following criteria at baseline: (1) participants with MetS, (2) one of the 6 indices (BMI, WC, Blood pressure, Hyperglycemia, TG, HDL-C) missing, and (3) Age/Sex/Educational level/Marital status/ Current residence/Current smoking/Alcohol drinking/Taking activities/Having regular exercises/Chronic diseases missing, a total of 9,588 participants who were aged 45 and above were included in our analyses after excluding missing data respondents, and without any direct interaction with people, all data were presented in the open as microdata at http://charls.pku.edu.cn/index/zh-cn.html. We obtained written informed consent from each of the participants, and the study was approved by the Ethics Committee of the China Center for Economic Research at Peking University.

### Data collection and measurement

The study subjects for this investigation were chosen from the China Health and Retirement Longitudinal Study (CHARLS), Wave 1 (2011) (22). The CHARLS Wave 2011 was used to select participants for this study for demographic characteristics and factors, such as age (four sections: 45–54, 55–64, 65–74, and above 75 years old), education (five sections: Illiterate, Less than elementary school, High school, Above vocational school), marital status (Single, or Married), current residence (Rural, or Urban), smoking (three sections: no, Former smoke, Current smoke) and alcohol using (three sections: no, Less than once a month, More than once a month), taking activity (two sections: no or yes)including interacting with friends/providing help to family, friends or neighbors who do not live with you/attending a sports, social or other type of club/playing mahjong, chess, cards; or going to a community club/participating in a community-related organization/doing volunteer or charity work/caring for an adult who is sick or disabled, who does not live with you and does not pay you/attending an education or training course/investing in stocks and shares/ Using the internet (23), having regular exercises (three sections: No exercise, Less than regular exercises, Regular exercises), regular physical exercise was defined as exercising at least 3 days per week and more than 30 min per day, including moderate to vigorous physical activity and walking and chronic disease counts (three sections:0, 1–2, 3–14) including (1) hypertension, (2) cancer or malignant neoplasm (excluding minor skin cancers), (3) diabetes mellitus or hyperglycemia, (4) chronic lung disease, (5) dyslipidaemia, (6) diseases of the liver (other than fatty liver, neoplasm, and cancer), (7) diseases of the kidneys (other than tumors or cancer), (8) stroke, (9) heart attack, coronary heart disease, angina pectoris, congestive heart failure, or other heart problems, (10) stomach or other digestive disorders (except tumors or cancers), (11) memory-related disorders, (12) emotional, nervous, or psychiatric problems, (13) arthritis or rheumatism, and (14) asthma as reported by the respondent (diagnosed by a doctor) (24).

The Chinese Center for Disease Control and Prevention (CDC) in Beijing received venous blood samples within 2 weeks of their departure from the Centers for Disease Control and Prevention (CDC) station, processed and ready for refrigeration. Fasting plasma glucose (FPG), triglycerides (TG) and high-density lipoprotein cholesterol (HDL-C) were measured using an enzyme colorimetric method. All samples were performed in the Youanmen Clinical Laboratory of Capital Medical University (25). Body mass index (BMI) was calculated by dividing the body weight (kg) by the square of the height (m^2^) (26–28). The waist circumference was measured at the end of expiration by standing straight with the feet 25–30 cm apart and measuring the horizontal position of the midpoint between the lower edge of the ribcage and the midpoint of the line connecting the upper iliac crest spine (29). Subjects rested for half an hour and then their blood pressure was measured in a quiet environment, blood pressure was measured on the subject’s left arm using an Omron TM HEM-7112 Monitor (Manufacturer: Omron Co., Ltd., Dalian, China) (30). Support the subject’s left arm comfortably, palm up, and take the average of three measurements at 45-s intervals.

### Diagnosing standard of MetS

According to the definition of IDF (2006), the diagnosis of MetS is based on central obesity (Chinese definition, the waist circumference (WC) for central obesity was ≥90 cm for men and WC ≥85 for women) plus any two or more of the following components: (1) Elevated blood pressure: systolic blood pressure (SBP) ≥130 mmHg and/or diastolic blood pressure (DBP) ≥85 mmHg or treatment of previously diagnosed hypertension; (2) Hyperglycemia: FPG ≥100 mg/dL or using antidiabetic medications or previously diagnosed type 2 diabetes; (3) High TG: TG ≥150 mg/dL or specific treatment for this lipid.

abnormality at high-density; (4) Low HDL-C: HDL-C < 40 mg/dL for men and < 50 mg/dL for women or specific treatment for this lipid abnormality (4, 11).

According to the ATP III (2005) definition, the diagnosis of MetS is based on three or more of the following abnormalities: (1) Central obesity (WC ≥90 cm for men and ≥ 80 cm for women); (2) Elevated blood pressure (BP): systolic blood pressure (SBP) ≥130 mmHg and/or diastolic blood pressure (DBP) ≥85 mmHg or on antihypertensive drug treatment in a patient with a history of hypertension; (3) Hyperglycemia: FPG ≥100 mg/dL or on drug treatment for elevated glucose; (4) High TG: TG ≥150 mg/dL or on drug treatment for elevated triglycerides; (5) Low HDL-C: HDL-C < 40 mg/dL for men and < 50 mg/dL for women or on drug treatment for reduced HDL (31, 32).

According to the definition of ATP III (2001), the diagnosis of MetS is based on three or more of the following abnormalities: (1) Central obesity (WC ≥102 cm for men and ≥ 88 cm for women); (2) Elevated blood pressure: systolic blood pressure (SBP) ≥130 mmHg and/or diastolic blood pressure (DBP) ≥85 mmHg or on antihypertensive drug treatment in a patient with a history of hypertension; (3) Hyperglycemia: FPG ≥110 mg/dL or on drug treatment for elevated glucose; (4) High TG: TG ≥150 mg/dL or on drug treatment for elevated triglycerides; (5) Low HDL-C: HDL-C < 40 mg/dL for men and < 50 mg/dL for women or on drug treatment for reduced HDL (33).

According to the definition of CDS (2004), the diagnosis of MetS is based on three or more of the following abnormalities: (1) Central obesity: BMI ≥25 kg/m^2^ (BMI was classified according to the recommendations of Working Group of Obesity in China, <18.5 kg/ m2 (underweight), 18.5–23.9 kg/m2 (normal range), 24–27.9 kg/m2 (overweight), ≥28 kg/m2 (obesity)); (2) Elevated blood pressure: systolic blood pressure (SBP) ≥140 mmHg and/or diastolic blood pressure (DBP) ≥90 mmHg or on antihypertensive drug treatment in a patient with a history of hypertension; (3) Hyperglycemia: FPG ≥110 mg/dL or on drug treatment for elevated glucose; (4) High TG: TG ≥150 mg/dL or on drug treatment for elevated triglycerides; (5) Low HDL-C: HDL-C < 40 mg/dL for men and < 50 mg/dL for women or on drug treatment for reduced HDL (34).

According to the WHO (1999) definition, (1) Central obesity (WC ≥90 cm or BMI ≥30 kg/m^2^ for men and ≥ 85 cm for women); (2) Elevated blood pressure: systolic blood pressure (SBP) ≥140 mmHg and/or diastolic blood pressure (DBP) ≥90 mmHg or on antihypertensive drug treatment in a patient with a history of hypertension; (3) Hyperglycemia: FPG ≥110 mg/dL or on drug treatment for elevated glucose; (4) High TG: TG ≥150 mg/dL or on drug treatment for elevated triglycerides; (5) Low HDL-C: HDL-C < 35 mg/dL for men and <39 mg/dL for women or on drug treatment for reduced HDL (35).

### Statistical analysis

We used Statistical Product Service Solutions (SPSS) software, version 22.0, to conduct the analyses (IBM SPSS, Armonk, NY, USA). The basic characteristics of the study population were expressed as number of cases and percentage (%), and the chi-square test was used to understand the distribution of categorical variables. Differences between groups for continuous and categorical variables were assessed using *t*-tests and *x*^2^ tests. The kappa value (kappa ≤0.20, poor; kappa = 0.21–0.40, fair; kappa = 0.41–0.60, moderate; kappa = 0.61–0.80, substantial; kappa > 0.80, very good) was used to assess the differences and consistency between the five definitions (36). Variables potentially associated with any defined MetS were assessed using binary and multivariable logistic regression, and those with *p* < 0.10 were included in multivariable logistic regression. Ratio ratios (ORs) and 95% confidence intervals (95% CIs) were calculated by sex for each of the five definitions of age, education, marital status, place of residence, current smoking, alcohol consumption, activity level, regular exercise, and chronic disease. Statistically significant, *p* < 0.05.

## Result

Table 1 shows the demographic characteristics of the subjects, which consisted of 9,588 subjects, of whom 4,444 (46.35%) were males and 5,144 (53.65%) were females. The overall mean age was 58.4 years with a standard deviation (SD) of 9.04, 59.3 years for males (SD = 8.78) and 57.5 years for females (SD = 9.17). The age range was 45–98 years. The majority (92.70%) lived in rural areas, and 62.61% had primary education or less, with 73.31% males and 53.36% females. The vast majority were married (89.18%) and a few were single (10.82%); 49.18% had one or two chronic diseases, and 60.51% of males were current smokers and 46.22% drinkers, while only 5.99% of females and 6.82% of females were current smokers and drinkers. Among them, the differences between males and females in terms of age, education level, marital status, current smoking, alcohol drinking, having regular exercises and number of chronic diseases were statistically significant (*p* < 0.05). However, the differences were not statistically significant (*p* > 0.05) in terms of current residence and taking activities.

**Table 1:** Characteristics of participants with full samples (*N* = 9,588).

Variables	Male	Female	Total	*X^2^*	*P*
	*N* (%)	*N* (%)	*N* (%)		
*N*	4,444 (46.35)	5,144 (53.65)	9,588		
Age (years)				84.503	0.000
45–54	1,378 (31.01)	2043 (39.72)	3,421 (35.68)		
55–64	1873 (42.15)	1980 (38.49)	3,853 (40.19)		
65–74	950 (21.38)	877 (17.05)	1827 (19.06)		
≥75	243 (5.47)	244 (4.74)	487 (5.08)		
Education				930.671	0.000
Illiterate	576 (12.96)	2069 (40.22)	2,645 (27.59)		
Less than elementary school	3,258 (73.31)	2,745 (53.36)	6,003 (62.61)		
High school	388 (8.73)	239 (4.65)	627 (6.54)		
Above vocational school	222 (5.00)	91 (1.77)	313 (3.26)		
Marital status				39.778	0.000
Single	385 (8.66)	652 (12.67)	1,037 (10.82)		
Married	4,059 (91.34)	4,492 (87.33)	8,551 (89.18)		
Current residence				0.002	0.965
Rural	4,119 (92.69)	4,769 (92.71)	8,888 (92.70)		
Urban	325 (7.31)	375 (7.29)	700 (7.30)		
Current smoking				4565.819	0.000
No	1,104 (24.84)	4,749 (92.32)	5,853 (61.05)		
Former smoke	651 (14.65)	87 (1.69)	738 (7.70)		
Current smoke	2,689 (60.51)	308 (5.99)	2,997 (31.26)		
Alcohol drinking				2354.714	0.000
No	1879 (42.28)	4,538 (88.22)	6,417 (66.93)		
Less than once a month	511 (11.50)	255 (4.96)	766 (7.99)		
More than once a month	2054 (46.22)	351 (6.82)	2,405 (25.08)		
Taking activities				0.268	0.605
No	2,252 (50.68)	2,634 (51.21)	4,886 (50.96)		
Yes	2,192 (49.32)	2,510 (48.79)	4,702 (49.04)		
Having regular exercises				7.764	0.021
No exercise	2,856 (64.27)	3,164 (61.51)	6,020 (62.79)		
Less than regular exercises	801 (18.02)	997 (19.38)	1798 (18.75)		
Regular exercises	787 (17.71)	983 (19.11)	1770 (18.46)		
Chronic diseases (counts)				17.296	0.000
0	1,523 (34.27)	1,602 (31.14)	3,125 (32.59)		
1–2	2,177 (48.99)	2,538 (49.34)	4,715 (49.18)		
3–14	744 (16.74)	1,004 (19.52)	1748 (18.23)		

Table 2 shows the prevalence of MetS according to the criteria of IDF (2006), ATP III (2005), ATP III (2001), CDS (2004), and WHO (1999). The overall population reveals a prevalence of MetS of 34.52% (22.32% among males and 45.06% among females) according to the standards of IDF (2006); 38.63% (27.99% among males and 47.82% among females) according to the rules of ATP III (2005); 25.94% (15.37% for males, 35.07% for females) according to the standards of ATP III (2001); 26.31% (19.96% for males and 31.80% for females) based on data from CDS (2004); and 21.57% (17.44% for males and 25.14% for females) according to WHO (1999). Based on the five definitions, there are subtle variations in the association between specific variables and MetS, although the similarity remains evident. Regardless of the definition used, having a primary school education or lower, being married, residing in rural areas, lack of exercise, and having one or two chronic diseases are significantly associated with higher prevalence rates among the general population (both genders). The prevalence of metabolic equivalent of task (MET) reaches its peak in the 55–64 age group among both genders of the general population, and decreases with age under all definitions. Regardless of the definition used, a higher prevalence of metabolic syndrome is associated with smoking in men, and with smoking a lower prevalence of metabolic syndrome is associated in women according to IDF (2006), ATP III (2005), and WHO (1999). Meanwhile, alcohol consumption shows a lower prevalence in women and a higher prevalence in men across ATP III (2005), CDS (2004), and WHO (1999).

**Table 2:** The prevalence of MetS defined by different definitions.

	**IDF (2006) %**			**ATP III (2005) %**			**ATP III (2001) %**			**CDS (2004) %**			**WHO (1999) %**		
	Male	Female	Total	Male	Female	Total	Male	Female	Total	Male	Female	Total	Male	Female	Total
Total	22.32	45.06	34.52	27.99	47.82	38.63	15.37	35.07	25.94	19.96	31.80	26.31	17.44	25.14	21.57
Age (years)															
45–54	35.89	36.24	36.13	33.44	35.81	35.02	33.24	35.86	35.14	35.74	37.59	36.94	34.58	33.26	33.75
55–64	42.64	40.16	40.91	43.17	39.84	40.96	43.78	39.30	40.53	44.19	38.75	40.67	44.77	40.76	42.26
65–74	17.54	18.68	18.34	18.25	18.90	18.68	17.86	19.35	18.94	16.23	18.89	17.95	16.77	20.11	18.86
≥75	3.93	4.92	4.62	5.14	5.45	5.35	5.12	5.49	5.39	3.83	4.77	4.44	3.87	5.88	5.13
*P*	0.000	0.000	0.223	0.007	0.000	0.404	0.084	0.000	0.795	0.000	0.057	0.084	0.000	0.000	0.130
Education															
Illiterate	8.17	39.43	30.06	9.41	40.04	29.75	10.10	39.86	31.68	9.47	39.00	28.62	9.42	40.22	28.68
Less than elementary school	73.39	54.06	59.85	72.99	53.54	60.07	72.33	54.16	59.15	73.73	55.32	61.79	73.55	54.06	61.36
High school	10.08	4.70	6.31	9.73	4.67	6.37	9.37	4.21	5.63	9.02	4.10	5.83	8.77	4.10	5.85
Above vocational school	8.37	1.81	3.78	7.88	1.75	3.81	8.20	1.77	3.54	7.78	1.59	3.77	8.26	1.62	4.11
*P*	0.000	0.775	0.000	0.000	0.994	0.000	0.000	0.672	0.000	0.000	0.202	0.073	0.000	0.688	0.024
Marital status															
Single	6.45	13.33	11.27	7.40	13.41	11.39	7.17	12.36	10.94	5.75	11.86	9.71	6.71	13.77	11.12
Married	93.55	86.67	88.73	92.60	86.59	88.61	92.83	87.64	89.06	94.25	88.14	90.29	93.29	86.23	88.88
*P*	0.005	0.201	0.299	0.061	0.127	0.149	0.133	0.619	0.821	0.001	0.229	0.037	0.033	0.173	0.613
Current residence															
Rural	88.10	91.76	90.66	88.59	91.75	90.69	88.73	91.80	90.95	89.40	91.81	90.96	89.03	91.65	90.67
Urban	11.90	8.24	9.34	11.41	8.25	9.31	11.27	8.20	9.05	10.60	8.19	9.04	10.97	8.35	9.33
*P*	0.000	0.018	0.000	0.000	0.011	0.000	0.000	0.064	0.000	0.000	0.090	0.000	0.000	0.089	0.000
Current smoking															
No	27.82	92.45	73.08	26.77	92.4	70.36	27.96	92.07	74.47	27.40	92.11	69.36	28.00	91.72	67.84
Former smoke	18.25	2.20	7.01	17.77	2.24	7.45	18.45	2.22	6.67	18.71	2.08	7.93	18.97	2.78	8.85
Current smoke	53.93	5.35	19.91	55.47	5.37	22.19	53.59	5.71	18.86	53.89	5.81	22.71	53.03	5.49	23.31
*P*	0.000	0.009	0.000	0.000	0.003	0.000	0.000	0.085	0.000	0.000	0.322	0.000	0.000	0.002	0.000
Alcohol drinking															
No	43.25	90.12	76.07	43.57	89.96	74.38	47.88	90.91	79.09	46.34	91.14	75.39	45.81	91.11	74.13
Less than once a month	10.99	4.18	6.22	11.01	4.27	6.53	9.37	4.38	5.75	10.94	4.28	6.62	9.29	3.87	5.90
More than once a month	45.77	5.69	17.70	45.42	5.77	19.09	42.75	4.71	15.16	42.73	4.58	17.99	44.90	5.03	19.97
*P*	0.731	0.001	0.000	0.529	0.001	0.000	0.004	0.000	0.000	0.023	0.000	0.000	0.029	0.001	0.000
Taking activities															
No	47.08	48.32	47.95	47.67	48.90	48.49	47.58	48.78	48.45	46.67	48.35	47.76	47.35	48.26	47.92
Yes	52.92	51.68	52.05	52.33	51.10	51.51	52.42	51.22	51.55	53.33	51.65	52.24	52.65	51.74	52.08
*P*	0.010	0.000	0.000	0.012	0.002	0.000	0.079	0.011	0.004	0.008	0.005	0.000	0.042	0.014	0.002
Having regular exercises															
No exercise	64.92	62.55	63.26	65.68	62.48	63.55	65.59	61.81	62.85	64.15	61.80	62.62	65.03	62.26	63.30
Less than regular exercises	18.45	17.43	17.73	17.60	17.60	17.60	17.86	17.85	17.85	18.94	18.34	18.55	18.45	18.10	18.23
Regular exercises	16.63	20.02	19.00	16.72	19.92	18.84	16.54	20.34	19.30	16.91	19.87	18.83	16.52	19.64	18.47
*P*	0.592	0.004	0.151	0.437	0.007	0.071	0.654	0.060	0.255	0.632	0.350	0.844	0.626	0.389	0.781
Chronic diseases (counts)															
0	26.01	24.94	25.26	27.57	25.24	26.03	24.60	22.23	22.88	23.68	20.84	21.84	24.00	18.10	20.31
1–2	49.60	50.09	49.94	48.31	50.28	49.62	47.44	50.55	49.70	48.37	51.04	50.10	48.26	51.43	50.24
3–14	24.40	24.98	24.80	24.12	24.47	24.35	27.96	27.22	27.42	27.96	28.12	28.06	27.74	30.47	29.45
*P*	0.000	0.000	0.000	0.000	0.000	0.000	0.000	0.000	0.000	0.000	0.000	0.000	0.000	0.000	0.000

MetS, metabolic syndrome; IDF, International Diabetes Federation; ATP III, Adult Treatment Panel III; CDS Chinese Diabetes Society; WHO World Health Organization.

Table 3 demonstrates consistency and variability in diagnoses using IDF (2006), ATP III (2005), ATP III (2001), CDS (2004) and WHO (1999) criteria. It also displays the kappa values between two definitions for males and females. Among males, only the agreement between IDF (2006) and ATP III (2001) (kappa = 0.51) was poor. The kappa values ranged from 0.64 to 0.85 with good agreement for any two other definitions. Among women, there was good agreement between IDF (2006) and both ATP III (2005) (kappa = 0.95) and ATP III (2001) (kappa = 0.71); ATP III (2005) also showed good agreement with ATP III (2001) (kappa = 0.74) and CDS (2004) (kappa = 0.67). Additionally, ATP III (2001) demonstrated good agreement with CDS (2004) (kappa = 0.85) and WHO (1999) (kappa = 0.71); and there was good agreement observed between CDS (2004) and WHO (1999) (kappa = 0.73). Moderate agreement was found between IDF (2006) and CDS (2004) (kappa = 0.44), and WHO (1999) (kappa = 0.55); as well as between ATP III (2005) and WHO (1999) (kappa = 0.54).

**Table 3:** The agreement between the various definitions of the MetS.

**MetS components**	**ATP III (2005)**		**ATP III (2001)**		**CDS (2004)**		**WHO (1999)**	
	**Kappa**	**95%CI**	**Kappa**	**95%CI**	**Kappa**	**95%CI**	**Kappa**	**95%CI**
**Male**								
**IDF (2006)**	0.85	0.83–0.87	0.51	0.48–0.54	0.68	0.65–0.71	0.73	0.70–0.75
**ATP III (2005)**			0.64	0.61–0.66	0.74	0.72–0.76	0.70	0.68–0.73
**ATP III (2001)**					0.76	0.73–0.78	0.68	0.65–0.71
**CDS (2004)**							0.80	0.77–0.82
**Female**								
**IDF (2006)**	0.95	0.94–0.95	0.71	0.69–0.73	0.44	0.41–0.46	0.55	0.52–0.57
**ATP III (2005)**			0.74	0.72–0.76	0.67	0.65–0.68	0.54	0.51–0.56
**ATP III (2001)**					0.85	0.84–0.87	0.71	0.69–0.73
**CDS (2004)**							0.73	0.71–0.75

MetS, metabolic syndrome, CI, confidence interval, IDF, International Diabetes Federation; ATP III, Adult Treatment Panel III; CDS, Chinese Diabetes Society; WHO, World Health Organization.

Table 4 analyzes the factors connected with MetS in men and women under different definitions. The study suggested that age, education, marital status, current residence, smoking, alcohol consumption, taking activities and number of chronic diseases were associated with MetS. However, the relationship between these factors varies by definition and by gender. Under all five definitions of MetS, being married, living in the city, and having a chronic disease were remarkably associated with a high prevalence of MetS in both male and female. Among men under any of the definitions, being 75 years of age and older was associated with a lower prevalence of MetS, while having a college degree and older was significantly associated with a higher prevalence of MetS. In contrast, among women, all ages 55 and older were conspicuously connected with a higher prevalence of MetS under all five definitions, however, education did not have a significant relationship in women. For men, smoking was obviously related to a lower prevalence of MetS in all five definitions, and not with MetS in women. For men, alcohol consumption was significantly associated with a lower prevalence of MetS only under the ATP (2001) and CDS (2004) definitions, and in women, alcohol consumption was significantly associated with a lower prevalence of MetS under the five definitions. In women, activity was significantly associated with a higher prevalence of MetS than MetS in men. Exercise was not associated with MetS in men and did not play a role in MetS in women.

**Table 4:** Factors related to MetS defined by IDF (2006), ATPIII (2005), ATPIII (2001), CDS (2004) and WHO (1999).

	IDF (2006)		ATP III (2005)		ATP III (2001)		CDS (2004)		WHO (1999)	
	Male	Female	Male	Female	Male	Female	Male	Female	Male	Female
Age (years)										
45–54	Reference	Reference	Reference	Reference	Reference	Reference	Reference	Reference	Reference	Reference
55–64	0.837 (0.712–0.985)^*^	1.271 (1.122–1.44)^***^	0.930 (0.798–1.083)	1.293 (1.142–1.464)^***^	0.963 (0.798–1.163)	1.204 (1.056–1.372)^**^	0.886 (0.749–1.048)	1.094 (0.957–1.250)	0.942 (0.789–1.124)	1.361 (1.176–1.574)^***^
65–74	0.644 (0.525–0.789)^***^	1.397 (1.191–1.637)^***^	0.726 (0.601–0.877)^**^	1.489 (1.270–1.745)^***^	0.747 (0.589–0.947)^*^	1.426 (1.210–1.681)^***^	0.598 (0.481–0.743)^***^	1.263 (1.068–1.494)^**^	0.657 (0.523–0.825)^***^	1.581 (1.321–1.892)^***^
≥75	0.549 (0.382–0.789)^***^	1.256 (0.962–1.639)	0.827 (0.608–1.125)	1.607 (1.230–2.098)^***^	0.853 (0.580–1.254)	1.473 (1.122–1.934)^**^	0.544 (0.371–0.799)^**^	1.091 (0.820–1.451)	0.583 (0.389–0.874)^**^	1.697 (1.268–2.270)^***^
Education										
Illiterate	Reference	Reference	Reference	Reference	Reference	Reference	Reference	Reference	Reference	Reference
Less than elementary school	1.620 (1.258–2.085)^***^	1.146 (1.016–1.291)^*^	1.455 (1.167–1.813)^**^	1.122 (0.996–1.264)	1.260 (0.959–1.656)	1.129 (0.996–1.278)	1.328 (1.034–1.707)	1.155 (1.017–1.313)^*^	1.342 (1.029–1.751)^*^	1.137 (0.991–1.304)
High school	1.784 (1.272–2.502)^**^	1.224 (0.929–1.613)	1.630 (1.200–2.215)^*^	1.232 (0.936–1.623)	1.330 (0.909–1.947)	1.026 (0.765–1.378)	1.227 (0.865–1.741)	0.953 (0.703–1.293)	1.225 (0.845–1.775)	1.048 (0.754–1.457)
Above vocational school	3.411 (2.376–4.896)^***^	1.123 (0.736–1.713)	2.996 (2.142–4.189)^***^	1.033 (0.677–1.576)	2.393 (1.611–3.555)^***^	1.059 (0.681–1.646)	2.427 (1.677–3.510)	0.916 (0.576–1.459)	2.599 (1.773–3.812)^***^	0.939 (0.569–1.547)
Marital status										
Single	Reference	Reference	Reference	Reference	Reference	Reference	Reference	Reference	Reference	Reference
Married	1.258 (0.947–1.670)	1.021 (0.855–1.219)	1.118 (0.872–1.434)	1.055 (0.884–1.260)	1.148 (0.836–1.575)	1.252 (1.039–1.509)^*^	1.468 (1.078–2.000)^*^	1.238 (1.021–1.501)*	1.208 (0.887–1.644)	1.085 (0.888–1.325)
Current residence										
Rural	Reference	Reference	Reference	Reference	Reference	Reference	Reference	Reference	Reference	Reference
Urban	1.867 (1.461–2.385)^***^	1.239 (0.993–1.547)	1.893 (1.496–2.396)^***^	1.288 (1.031–1.608)^*^	1.637 (1.241–2.159)^***^	1.257 (1.012–1.561)^*^	1.573 (1.215–2.038)^***^	1.232 (0.988–1.536)	1.607 (1.230–2.099)^***^	1.242 (0.983–1.569)
Current smoking										
No	Reference	Reference	Reference	Reference	Reference	Reference	Reference	Reference	Reference	Reference
Former smoke	1.214 (0.972–1.516)	1.621 (1.052–2.499)^*^	1.227 (0.995–1.514)	1.720 (1.106–2.677)^*^	1.177 (0.915–1.512)	1.480 (0.965–2.272)	1.285 (1.023–1.616)^*^	1.339 (0.865–2.073)	1.244 (0.980–1.579)	1.930 (1.250–2.982)^**^
Current smoke	0.768 (0.649–0.908)^**^	0.796 (0.628–1.008)	0.825 (0.705–0.965)^*^	0.781 (0.617–0.987)^*^	0.773 (0.637–0.937)^**^	0.903 (0.706–1.155)	0.779 (0.654–0.928)^**^	0.950 (0.739–1.221)	0.751 (0.625–0.903)^**^	0.853 (0.648–1.124)
Alcohol drinking										
No	Reference	Reference	Reference	Reference	Reference	Reference	Reference	Reference	Reference	Reference
Less than once a month	0.885 (0.695–1.126)	0.728 (0.561–0.944)^*^	0.887 (0.710–1.108)	0.748 (0.579–0.968)^*^	0.669 (0.500–0.894)^**^	0.813 (0.619–1.068)	0.792 (0.617–1.017)	0.784 (0.591–1.040)	0.681 (0.516–0.898)^**^	0.712 (0.518–0.977)^*^
More than once a month	0.941 (0.807–1.099)	0.701 (0.560–0.877)^**^	0.928 (0.804–1.070)	0.705 (0.564–0.880)^**^	0.783 (0.657–0.934)^**^	0.558 (0.434–0.719)^***^	0.780 (0.665–0.916)^**^	0.554 (0.426–0.720)^***^	0.864 (0.731–1.021)	0.638 (0.483–0.842)^**^
Taking activities										
No	Reference	Reference	Reference	Reference	Reference	Reference	Reference	Reference	Reference	Reference
Yes	1.107 (0.958–1.280)	1.246 (1.115–1.392)^***^	1.096 (0.958–1.253)	1.211 (1.084–1.353)^***^	1.104 (0.935–1.305)	1.191 (1.060–1.337)^**^	1.172 (1.008–1.363)^*^	1.204 (1.069–1.355)^**^	1.115 (0.952–1.307)	1.197 (1.054–1.359)^**^
Having regular exercises										
No exercise	Reference	Reference	Reference	Reference	Reference	Reference	Reference	Reference	Reference	Reference
Less than regular exercises	1.038 (0.859–1.254)	0.813 (0.703–0.940)^**^	0.958 (0.803–1.144)	0.824 (0.713–0.952)^**^	0.983 (0.790–1.224)	0.887 (0.761–1.033)	1.072 (0.882–1.304)	0.922 (0.789–1.077)	1.028 (0.836–1.264)	0.914 (0.773–1.082)
Regular exercises	0.889 (0.731–1.083)	1.032 (0.893–1.193)	0.868 (0.725–1.041)	1.031 (0.892–1.192)	0.882 (0.704–1.105)	1.077 (0.927–1.252)	0.930 (0.759–1.141)	1.031 (0.884–1.203)	0.896 (0.722–1.111)	1.005 (0.851–1.185)
Chronic diseases (counts)										
0	Reference	Reference	Reference	Reference	Reference	Reference	Reference	Reference	Reference	Reference
1–2	1.465 (1.236–1.736)^***^	1.466 (1.288–1.669)^***^	1.323 (1.133–1.544)^***^	1.458 (1.282–1.658)^***^	1.413 (1.156–1.727)^***^	1.646 (1.431–1.894)^***^	1.558 (1.298–1.869)^***^	1.811 (1.564–2.097)^***^	1.521 (1.256–1.842)^***^	2.015 (1.707–2.378)^***^
3–14	2.535 (2.057–3.125)^***^	2.311 (1.963–2.721)^***^	2.415 (1.990–2.931)^***^	2.231 (1.895–2.626)^***^	2.833 (2.242–3.581)^***^	2.730 (2.304–3.234)^***^	3.361 (2.706–4.174)^***^	3.058 (2.569–3.641)^***^	3.102 (2.475–3.888)^***^	3.561 (2.946–4.305)^***^

*P < 0.05, **p < 0.01, ***p < 0.001, OR (95%CI), calculated with multivariable logistic regression stratified by sex. MetS, Metabolic syndrome; OR, Odds Ratio; CI, Confidence Interval; IDF, International Diabetes Federation; ATP III, Adult Treatment Panel III; CDS, Chinese Diabetes Society; WHO, World Health Organization. Factors in the model: age, education, marital status, current residence, current smoking, alcohol drinking, taking activities, having regular exercises, chronic diseases.

## Discussion

This study has found that the binary logistic regression showed that age, education, marital status, current residence, current smoking, alcohol drinking, having regular exercises and number of chronic diseases were associated with MetS. However, the strength of this association varied by gender and definition. Examining the relationship between these factors and MetS will help in better management of MetS; therefore, understanding the differences between definitions will help correctly analyze the differences in the prevalence of MetS. Studies have shown that the prevalence in our population is consistent with previous Chinese data (37, 38). In a study of people 45 years and older, the prevalence of MetS was 38.4 and 39.7% according to IDF (2006) and ATP III (2005), respectively (39). In a study of people 35 years and older, the prevalence of MetS was 18.57% according to the criteria of CSD (2004) (40), and in a study of people 20 years and older, the prevalence of MetS was 14.7% according to the criteria of World Health Organization (1999) (41). The study showed a significantly higher prevalence of MetS according to the ATP III (2005) diagnostic criteria. This indicates that the ATP III (2005) criteria are more stringent than the other criteria.

Previous studies have shown that the prevalence of MetS increases with age, with gender differences (42). In the current study, the prevalence of MetS climbed to a peak at the age of 55–64 years, which is close to the findings of the study that peaked at the age of 60–69 years (43). In addition to this, gender also has a different effect on the prevalence of MetS, with age of the females significantly associated with a high prevalence of MetS, which may be related to menopause in women, where hormones in the body are reduced and lose their protective effect on the heart and kidneys, which may lead to increased blood pressure and a sharp increase in cardiovascular disease. In this study, the prevalence of MetS in men peaked at 55–64 years of age and then declined, becoming a protective factor above 65 years of age, which may be related to the fact that men tend to refuse or die before 75 years (16, 44).

In addition to sex and age, there are a number of other factors associated with MetS. The association of education with MetS varies, being negatively associated with men and not so much with women. This is consistent with males in a Korean study (45). The possible explanation for this is that the number of females in this study was higher in rural areas and in this era, females had less access to education than males. Men, on the other hand, have improved living conditions with higher education and were more tended to consume high-calorie foods while engaging in low physical work. Individuals who are married and live in urban areas have a higher risk of MetS, and it has been shown that this may be related to the fact that married people, as well as those living in urban areas, are more economically developed compared to rural areas, where there has been a dramatic increase in high-fat, high-purine fast food (46), and that married people may increase their intake of high-fat, high-purine foods due to their sense of well-being. In this study, according to the classification used (24), chronic diseases mainly include 14 types of disease, having chronic diseases were a high risk factor for MetS, and more attention should be given individuals with chronic diseases. The results of this study also showed that there was a significant negative correlation between smoking and MetS in men and not significant with MetS in women. The possible explanation for this is that the number of smokers in females is lower and the detection is not strong enough, and in males, due to the metabolic effects of nicotine in cigarettes, leading to weight loss in smokers, thereby affecting the diagnosis of MetS (47). In the IDF (2006), ATP III (2005), and WHO (1999) definitions, former smokers were significantly associated with MetS in women, whereas the association was not significant in men. In addition, we found two controversial results, alcohol consumption as a protective factor in women and no significant correlation in men. This is consistent with the results of Huang (16), Huang et al. demonstrated that alcohol consumption has a protective effect for women and a risk factor for men. Sun’s study also found that heavy alcohol consumption (>35 g/day) is associated with a high prevalence of MetS. According to relevant guidelines (13), small amounts of alcohol consumption can increase HDL-C levels, which may lower the risk of cardiovascular disease. The study found that alcohol consumption among women was a protective factor, possibly due to the low number of women who consume alcohol (88.22% of women in this study did not drink alcohol) and the small amount of alcohol consumed. Walker et al. demonstrated that small amounts of alcohol consumption have a cardioprotective effect on the heart (48). Whereas alcohol consumption was not significantly associated among males, this may be related to the fact that the male drinkers in this study were relatively small and alcohol consumpted relatively low due to limited economic conditions in that era. Another is that taking activity is a risk factor in women and not significant in men. The possible reason for this was that the activities in this study were not based on physical exertion and were mainly recreational activities that do not reduce the prevalence of MetS and further research is needed (23).

The purpose of this study was to investigate the prevalence and characteristics of five different definitions of metabolic syndrome (MetS) in China. The results of the analysis showed that the overall prevalence of MetS in the Chinese population aged 45 years and older, as defined by IDF (2006), ATP III (2005), ATP III (2001), CDS (2004) and WHO (1999), was 34.52, 38.63, 25.94, 26.31, and 21.57%. The results of our study showed that the prevalence of MetS was lower in men than in women in all definitions. This may be related to the fact that the indicators were higher in females than in males in this study. The prevalence and attributes of metabolic syndrome (MetS) in China varied greatly between definitions, ranging from 21.57 to 38.63%. Therefore, it is necessary to implement targeted interventions for different categories of metabolic syndrome.

There are limitations to our study: firstly, several definitions were compared, but they were less consistent among women, which may confuse researchers; secondly, this study was cross-sectional and did not adequately investigate the causal relationship between various MetS correlates; thirdly, factors could not be concluded due to the insufficient sample size, and some potentially relevant factors were not included in the study; fourthly, we did not have data on passive smokers in our study to conduct passive smoker exposure analyses; finally, this study included a significant number of middle-aged and older adults. However, only the surviving subjects were analyzed, which may have introduced bias into our preliminary findings. As well as the fact that our sample years may be too lengthy, further survey data is necessary for additional analysis. There are some strengths to this study. Firstly, the sample size was large, with 9,588 participants included, enabling a better estimation of MetS prevalence. Furthermore, to ensure accurate data, lipid and blood glucose measurements were taken at the Capital Medical University Youanmen Clinical Laboratory. Thirdly, we used five distinct definitions of MetS, which allows comparisons with data from other regions and offers a more comprehensive overview of the prevalence and features of MetS.

## Conclusion

According to the retrospective study, the prevalence of MetS varied when different definitions were used. The highest prevalence of MetS was 38.36% using the ATP III (2005) definition, followed by IDF (2006) (34.52%), CDS (2004) (26.31%), ATP III (2001) (25.94%), and WHO (1999) (21.57%). The agreement between the five definitions was good in men, but in women there was a large variability between CDS (2004) and IDF (2006), WHO (1999) and IDF (2006), and WHO (1999) and ATP III (2005). The prevalence of MetS in the Chinese population differs when different definitions are used, so it is necessary to investigate the reasons for the differences in the prevalence of MetS under different definitions and the factors associated with them.

## Data availability statement

The original contributions presented in the study are included in the article/supplementary material, further inquiries can be directed to the corresponding author.

## Author contributions

KM: Writing – original draft. HL: Writing – review & editing. LG: Writing – review & editing. JLL: Writing – review & editing. YL: Writing – review & editing. XL: Writing – review & editing. LS: Writing – review & editing. LY: Writing – review & editing. TY: Writing – review & editing. CW: Writing – review & editing. DZ: Writing – review & editing. JL: Writing – review & editing. ML: Writing – review & editing. YH: Writing – review & editing. LZ: Writing – review & editing.
